# Novel COVID-19: A Comprehensive Review of Transmission, Manifestation, and Pathogenesis

**DOI:** 10.7759/cureus.8184

**Published:** 2020-05-18

**Authors:** Azhar Hussain, Jasndeep Kaler, Elsa Tabrez, Salma Tabrez, Shams S.M. Tabrez

**Affiliations:** 1 Healthcare Administration, Franklin University, Columbus, USA; 2 Medicine, Xavier University School of Medicine, Oranjestad, ABW; 3 Internal Medicine, American University of Integrative Sciences, Bridgetown, BRB; 4 Family Medicine, Bay Area Family Practice, Orlando, USA; 5 Gastroenterologist and Hepatologist, University of Central Florida College of Medicine, Orlando, USA

**Keywords:** covid-19, sars-cov2, sars, mers, angiotensin converting enzyme 2, wuhan-hu-1-cov

## Abstract

A global outbreak highlights the start of a new decade as a new strain of coronaviruses emerges. Coronavirus disease 2019 (COVID-19), also referred to as Wuhan-Hu-1-CoV - amongst many other names - emerged from the West District of Southern China Seafood Wholesale Market in late December 2019. With the emergence of the new decade, the causative agent of COVID-19 was identified: severe acute respiratory syndrome coronavirus 2 (SARS-CoV2). COVID-19 became declared a global pandemic by the World Health Organization (WHO). COVID-19, currently, is affecting 204 countries and territories and two international conveyances. Initial stages of COVID-19 present with symptoms that mimic the common cold and individuals may be asymptomatic carriers and thus, transmitting the virus to others. COVID-19, like other coronaviruses, presents with S glycoproteins on the membrane that plays an integral role in the virus binding with the angiotensin-converting enzyme 2 (ACE2) receptor. The ACE2 receptor is an intramembrane receptor on the type II pneumocytes, where the virus is able to replicate after getting endocytosed within the cytoplasm. As the viral load increases within the alveolar cell, the alveolar epithelial cell will burst, releasing the newly replicated viral RNA. Elderly individuals are at a greater risk of infection due to weakened immune systems and pre-existing medical conditions resulting in a compromised immune response, also increasing the susceptibility of infection. Infected individuals presenting with mild to moderate symptoms are recommended to self-isolate as the majority will recover without any intervention.

## Introduction and background

For the third time in two decades, an outbreak has been linked back to the family of coronaviruses, causing a global pandemic leaving many countries in a state of despair.

**Figure 1:**
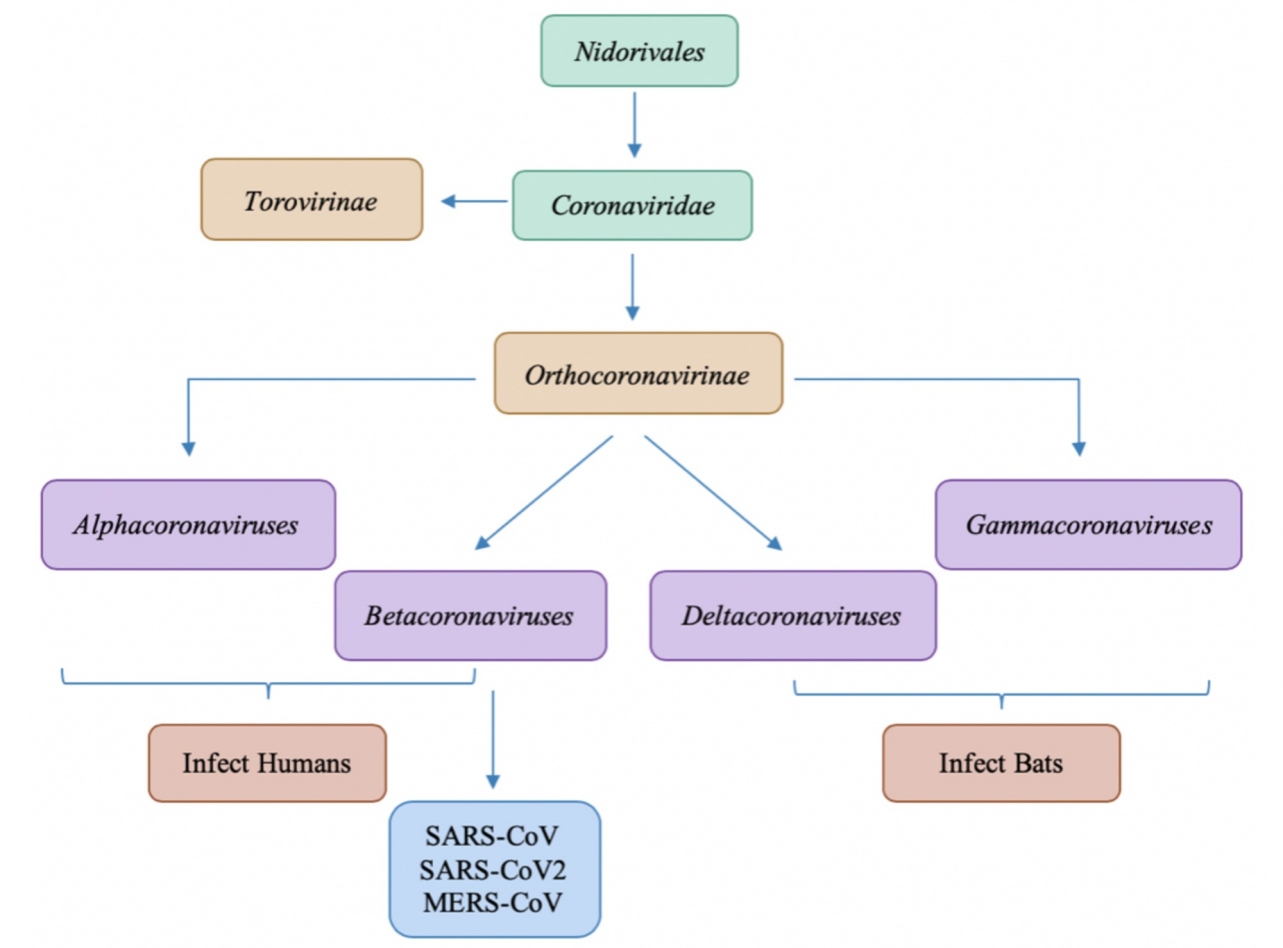
Differentiation of Coronaviruses within Nidorivales lineage

Figure 1 presents with a lineage of the Coronaviridae family that shows the different subcategories present. Taxonomically, the family Coronaviridae is classified into two subfamilies, the Orthocoronavirinae and the Torovirinae, with Orthocoronavirinae being further classified into four genera: Alphacoronavirus, Betacoronavirus, Deltacoronavirus and Gammacoronavirus, categorized in the order Nidovirales [1-2]. Research shows that though coronaviruses are widespread and though the widest varieties of genotypes infect bats, two subtypes infect humans: alphacoronaviruses and betacoronaviruses [3].

Coronaviruses (CoVs) are enveloped viruses with a large plus-strand RNA genome with its RNA genome being 27-32 kb in size, capped, and polyadenylated and range from 80 to 160 nm in diameter [4-5]. The genome of CoVs presents with a 5’-cap structure and 3’ poly-A tail [6]. Coronaviruses present with prominent surface projections that are up to 20 nm in length and cover the entire surface of the virion, giving them the corona appearance [5]. Two-thirds of the viral RNA, mainly located in the first open reading frame (ORF 1a/b), encode for 16 non-structure proteins (NSPs) and the rest of the virus genome encodes for essential structural proteins [7]. In the genomes of coronaviruses, insertion or recombination facilitates the acquisition of transforming low pathogenicity into highly pathogenic forms for polybasic cleavage sites [2]. Coronaviruses could have adopted a natural evolutionary mechanism to mutate and attain the polybasic cleavage site because the viruses must have both the mutations and the polybasic cleavage site for appropriate human ACE2 receptor binding [2,7]. There is a possibility that SARS-CoV2 ancestors transferred into humans, by attaining genetic features via adaptations leading to the current pandemic with COVID-19.

**Figure 2:**
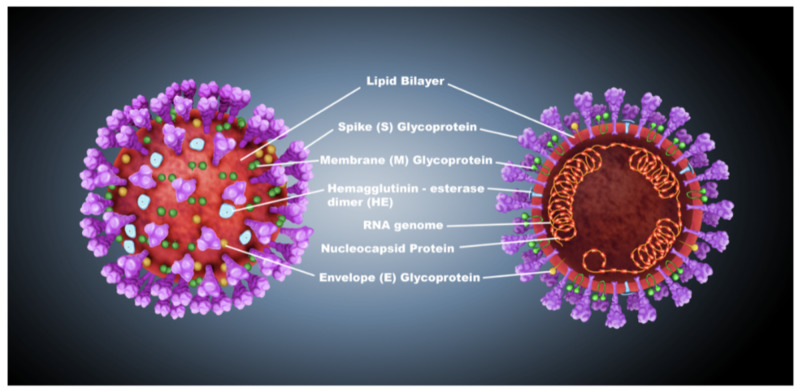
Structural proteins of coronavirus

Figure 2 depicts the essential structural proteins that are present on the coronavirus membrane.

The membrane (M) glycoprotein is the most abundant structural protein on the membrane of coronavirus; it spans the membrane bilayer three times, leaving a short NH2-terminal domain outside the virus and a long -COOH terminus, inside the virion, as a cytoplasmic domain [8]. The spike protein, a type I membrane glycoprotein that constitutes peplomers, plays an integral role in the initiation of viral infectivity [5,8]. The envelope (E) protein has been detected as a minor structural component and most likely constitutes the smaller spikes that are observed on the virus particles in some electron micrographs of coronaviruses [5,9]. In some betacoronaviruses, the presence of the hemagglutinin-esterase (HE) protein that is similar in function to influenza virus hemagglutinin allows the virus to bind to sialic acid on the host cell-surface glycoproteins [10]. Molecular interactions between the envelope proteins are thought to determine the formation and composition of the coronaviral membrane [9]. Figure 2 also denotes a nucleocapsid (N) protein that is present on the coronavirus membrane, and this protein is a phosphoprotein that binds to virion RNA and presents a regulatory function for viral RNA synthesis [5].

All the structural proteins were well conserved, except for spike glycoproteins that showed a high rate of mutation in coronavirus disease 2019 (COVID-19) [11]. Results demonstrate that compared with severe acute respiratory syndrome coronavirus (SARS-CoV), severe acute respiratory syndrome coronavirus 2 (SARS-CoV2) shares approximately 81% amino acid similarity in spike (S) protein, which represents less conserved patterns of S protein than other CoVs [12]. Despite the structural similarities amongst the coronavirus family, the three largest outbreaks in the last decade have been associated with betacoronaviruses subtype. A thorough genome analysis conducted to reveal that SARS-CoV2 (also known as COVID-19) was a descendant of Bat SARS/SARS-like CoVs and that bats served as a natural reservoir [2].

Severe acute respiratory syndrome-associated coronavirus (SARS-CoV) was first noted in the Guandong province of China in November 2002 and resulted in over 8,000 cases and approximately 750 deaths worldwide over the next several months until the end of the outbreak in July 2003 [13]. Severe acute respiratory syndrome coronavirus (SARS-CoV) typically presented with fever and symptoms of lower respiratory tract infection with radiographic evidence of pneumonia or acute respiratory distress syndrome (ARDS) [3]. The incubation period of SARS-CoV is between 2 and 20 days [3, 13]. Treatments attempted included corticosteroids and ribavirin which were not found to be beneficial; thus, supportive care remains the cornerstone of care for SARS-CoV [3].

Middle Eastern respiratory syndrome coronavirus (MERS-CoV) was first reported in September 2012 and since then, more than 2,400 cases of MERS-CoV have been reported to the World Health Organization (WHO) in and around the Arabian Peninsula [14]. Similar to SARS-CoV, presentation is typically fever with symptoms of lower respiratory tract infection and radiographic evidence of pneumonia or ARDS; other manifestations might include renal failure, anorexia, nausea, vomiting, diarrhea, abdominal pain, and disseminated intravascular coagulation [3]. The incubation period of MERS-CoV ranges from 1 to 14 days [3, 14]. Treatment for MERS-CoV was also supportive, focusing on the management of complications of sepsis and ARDS in intensive care units [3].

**Figure 3:**
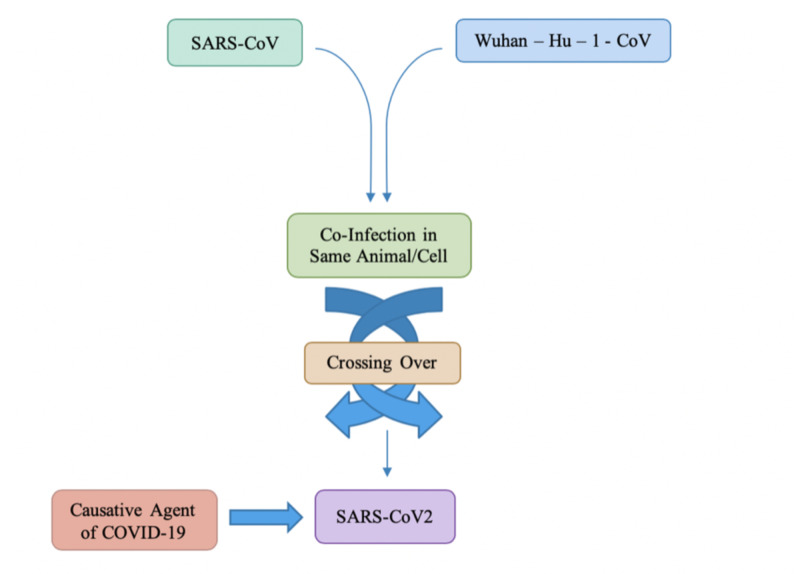
Crossing over due to co-infection causing COVID-19

The origin of COVID-19 can be traced back to the West District of Southern China Seafood Wholesale Market, located in Wuhan, a city in the province of Hubei [15]. This outbreak was initially referred to as Wuhan-Hu-1-CoV and later became known as COVD-19. Initial reports of COVID-19 suggest an incubation period similar to the incubation period of SARS-CoV and MERS-CoV and the clinical features are also rather similar to these viruses: fever, cough, chest tightness, dyspnea and difficulty breathing [3]. The similarity in symptoms between SARS-CoV and COVID-19 also stems from both viruses sharing the same receptor - angiotensin-converting enzyme receptor (ACE2) in the lungs. As illustrated in Figure 3, the causative agent of COVID-19 is the viral genome of SARS-CoV2.

This manuscript aims to identify the transmission of COVID-19 and the viral lifecycle including its replication, manifestation, and pathogenesis.

History & epidemiology

Human coronaviruses, first characterized in the 1960s, are responsible for a substantial proportion of upper respiratory tract infections in children [16]. Bats are known to be reservoir hosts for several human viruses, including rabies, Marburg, Nipah, Hendra, and severe acute respiratory syndrome coronavirus (SARS-CoV) [17]. Based on the large number of infected people that were exposed to the wet animal market in Wuhan City where live animals are routinely sold, the zoonotic origin of COVID-19 is suggested [18]. Both SARS-CoV2 and SARS-CoV originated from bats and likely transferred to humans via an intermediate host - pangolins and palm civets, respectively [19]. COVID-19 follows a person-to-person transmission that occurs primarily via direct contact or through droplets spread by coughing or sneezing from an infected individual [3,18]. COVID-19 has currently affected 212 countries and territories around the world and 2 international conveyances: The Diamond Princess cruise ship harbored in Yokohama, Japan, and Holland America’s MS Zaandam cruise ship [20]. According to Situation Report-112 published by WHO, as of May 11, 2020, there are 4,006,257 cases and 278,892 deaths, globally [21].

When looking at the transmission of COVID-19, it is important to discuss the concept of R0. R0 is referred to as the reproductive ratio, or in other words, the degree of transmissibility. R0 provides a representation of how many people can be infected by one person. The R0 for COVID-19 is roughly between 2 to 4 people [22]. This means that each infected individual possesses the ability to infect 2 to 4 other people. By social distancing and quarantining, an individual is actively avoiding the spread of respiratory droplets and by doing this, one could potentially help with decreasing the transmissibility of the virus.

## Review

The virus responsible for COVID-19 (also referred to as SARS-CoV2) is part of the SARS-like coronaviruses species and is almost certainly a descendant from a bat coronavirus, specifically closest to the Rhinolophus bat which is >96% homologous with the current SARS-CoV2 virus, in terms of the genome [23]. COVID-19 has a different coronavirus-specific nucleic acid sequence from known human coronavirus species, which are similar to some of the betacoronaviruses identified specifically in bats [24]. Pangolins are an endangered ant-eating mammal from which researchers in the city of Guangzhou have acquired a coronavirus with 99% genomic homology, with a receptor-binding domain identical to that of SARS-CoV2 [23]. Though the current SARS-CoV2 shares 79% of its genome with SARS-CoV, it is much more transmissible, as the mutations to the spike glycoproteins allow increased ability of SARS-CoV2 to attach onto ACE2 receptors [25].

The similarity between SARS-CoV and Wuhan-Hu-1-CoV (now more readily known as SARS-CoV2 or COVID-19) suggests the possibility of a co-infection of two CoVs in the same animal or cells that can potentially facilitate crossing over [2], as depicted in Figure 3.

In the S protein, the recombination event is certainly significant as it permits the virus to modify superficial antigenicity to get from the immune reconnaissance into the animals, and then to adapt its variations in order to infect a human host [2,25]. Research has confirmed that mutations in different genomic regions of SARS-CoV2 (COVID-19) have a specific influence on virus reproductive ability, allowing genotypes to adjust and quickly adapt in a rapidly changing environment [2].

Transmission & life cycle

Like many respiratory viruses, COVID-19 can be spread through tiny droplets released from the nose and mouth of an infected individual as they cough or sneeze [26-27]. As an estimate, a single cough is said to produce up to 3,000 droplets [27]. These droplets not only land on the individuals around the infected patient but will also land on several types of surfaces, and depending on the type of the surface, the virus is able to withhold through for a certain number of hours. The virus can be spread if an individual is to touch a contaminated object or surface and then touches their mucosal membranes such as the nose or the mouth [28]. Research has shown that the coronaviruses can be inactivated within a minute by disinfecting surfaces with 62% to 71% alcohol [27]. The exact duration of the life of the virus on different surfaces is yet to be determined due to a vast amount of speculation.

**Figure 4:**
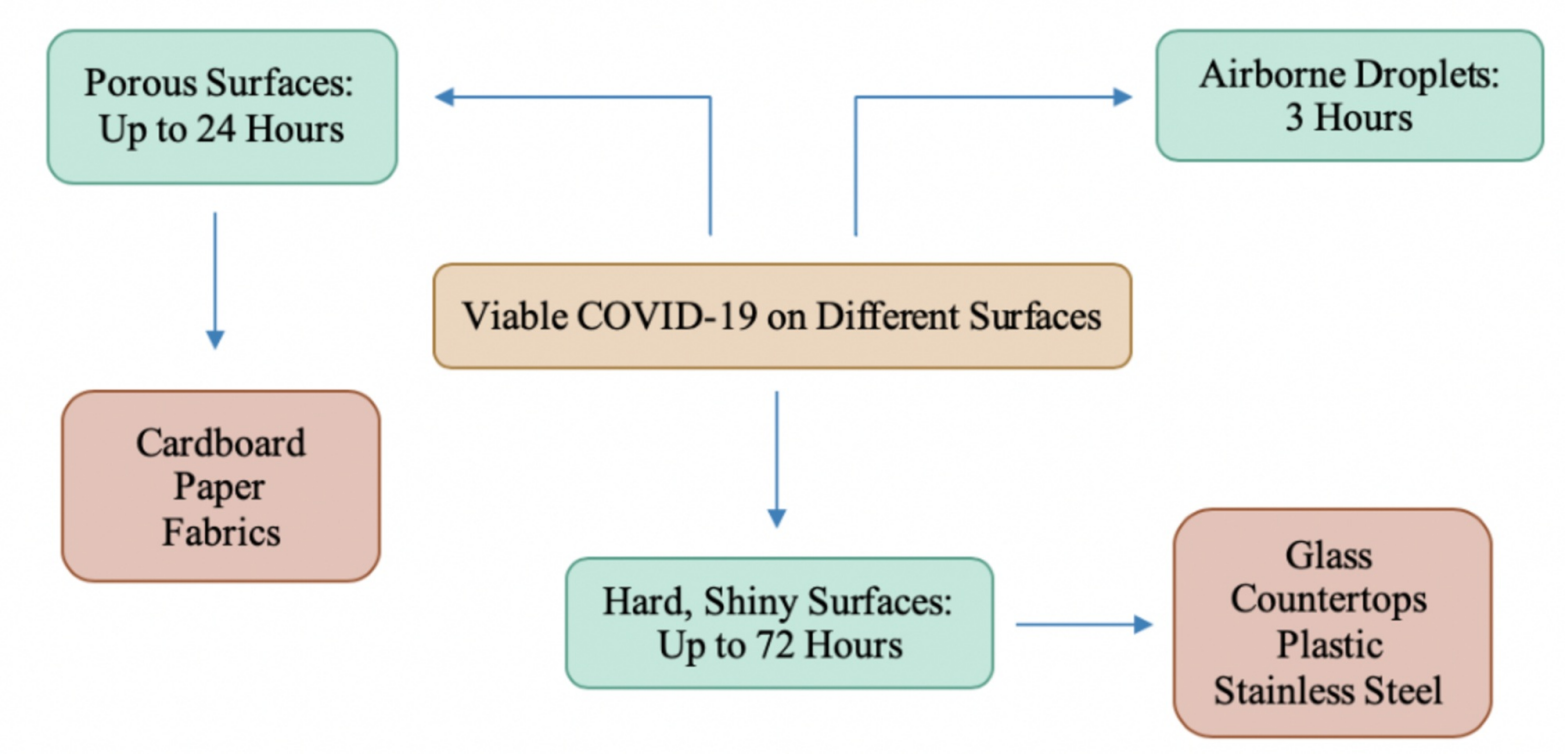
The survival time of coronavirus on different surfaces

Figure 4 depicts how long the virus remains on various surfaces.

Research suggests that a viable virus is able to be detected on stainless steel and plastic surfaces for up to 3 days, on cardboard for up to 24 hours, and on copper for 4 hours [28]. While the different types of surfaces have been investigated, there is no consensus on the temperature at which the virus is considered to be viable, along with the amount of virus necessary on these surfaces to be able to infect via fomite transmission. Researchers found SARS-CoV2 remains infectious in airborne droplets for at least 3 hours [29]. This causes speculation about how the risk can be reduced. Although fomite transmission is not deemed to be the major cause of transmission of COVID-19, it is important to minimize contact with such surfaces and to practice proper handwashing techniques. Although hand sanitizers and alcohol wipes can be used to sterilize the surfaces in cases where avoidance is not an option, it is important to continuously wash your hands and to avoid constantly touching the mucous membranes that lead to your airways [28-29].

As mentioned previously, SARS-CoV2 is transmitted via respiratory droplets through coughing/sneezing and/or by direct contact with the infected individual. Once the virus enters the host via the mucosal membranes, it travels through the respiratory tract and enters the lung alveoli. Inside the alveoli, the SARS-CoV2 binds specifically to the angiotensin-converting enzyme 2 (ACE2) receptor, present on the type II pneumocyte. There is an S glycoprotein present on the outer membrane of the SARS-CoV2 which has a high affinity for the ACE2 receptor [26]. Once the viral particle binds to the ACE2 receptor, the virus is endocytosed into the cytoplasm of the type II pneumocyte where the lysosomal enzymes of the host cell will break down the lipid bilayer of the virus. SARS-CoV2 will use ribosomes of the host cell and translate mRNA into polyproteins. These polyproteins are the structural framework of the viral molecule [30-31]. The synthesized polyproteins will utilize the host RNA-dependent RNA polymerase and start to replicate itself, increasing the viral load within the host cell. The polyproteins will use host enzymes such as proteinases, which will proteolyze the polyproteins to create the individual spike proteins, E protein, nucleocapsid, etc. [32]. These structural components, along with the replicated ssRNA, will form a mature SARS-CoV2 that will bud off the type II pneumocyte, back into the alveolus [7]. 

The type II pneumocytes are destroyed in the process of SARS-CoV2 budding off of the cell. As the type II pneumocytes are destroyed, they will release specific inflammatory mediators which will stimulate macrophages. Stimulated macrophages release specific cytokines such as interleukin-1 (IL-1), interleukin-6 (IL-6), and tissue necrosis factor-alpha (TNF-α) [33]. IL-1 and IL-6 are cytokines released in cases of acute inflammation, leading to fever [18]. Furthermore, IL-1, IL-6, and TNF-α will enter the bloodstream and cause smooth muscle dilation along with contraction of blood vessel endothelial cells, collectively increasing capillary permeability. As capillary permeability increases, plasma from the bloodstream will leak into the interstitial spaces, causing alveolar edema. As the type II pneumocytes within alveoli become destroyed by direct damage by SARS-CoV2, the surfactant production starts to decrease as well since the primary function of type II pneumocyte is surfactant production. Surfactant, which normally decreases the surface tension within alveolus, will cause alveolar collapse, due to increased surface tension, as well as alveolar edema [32]. This collapse of the alveoli will impair the gas exchange, leading to refractory hypoxemia. With decreased gas exchange, work associated with breathing increases largely due to attempting to breathe in as much air as possible to not only reopen the collapsed alveolus but also to open against the interstitial edema. This is the mechanism that leads to acute respiratory distress syndrome (ARDS). As more information comes to light about COVID-19, another feared complication that has been noted, alongside ARDS, has been disseminated intravascular coagulation. Reported in 71% of nonsurvivors, disseminated intravascular coagulation (DIC) is a manifestation of coagulation failure and an intermediate link in the development of multi-organ failure [34].

Inflammatory mediators released by the destruction of type II pneumocytes will cause a neutrophil influx into the alveolus. Through the release of reactive oxygen species and proteases, neutrophils will attempt to destroy the virus. While attempting to destroy the virus, neutrophils will cause mass destruction of all alveolar cells; type I and type II pneumocytes. Destroyed type I pneumocytes will be caused by the impaired gas exchange as these pneumocytes have an integral role in gas exchange. Type II pneumocytes are also destroyed by the neutrophils which, as previously mentioned, causes an increase in surface tension, leading to alveolar collapse. Destroyed cells will slough off the alveolar basement membrane into the center of the alveolus creating a collection of fluid, along with cellular debris consisting of type I pneumocytes, type II pneumocytes, neutrophils, and macrophages, leading to consolidation. The consolidation will also hinder gas exchange, causing hypoxemia [18]. Peripheral chemoreceptors will be triggered by hypoxemia causing the sympathetic nervous system (SNS) to increase respiration rate and heart rate to compensate for a decreased partial pressure of oxygen. Consolidation will cause a productive cough and may also cause the presentation of dyspnea due to decreased gas exchange.

Inflammation within the lungs can progress to systemic inflammatory response syndrome (SIRS) as cytokines circle through the vascular system, causing increased capillary permeability [35]. Following the same mechanism as in the lungs, the increased permeability will cause the plasma to deposit within tissue spaces, decreasing the blood volume. The vasodilation of blood vessels will decrease the total peripheral resistance (TPR), causing a significant drop in blood pressure. The hypotension will cause decreased perfusion, leading to multi-system organ failure (MSOF) [33]. Decreased perfusion to kidneys will cause an increase in blood urea nitrogen (BUN) and creatinine. Kidneys are unable to filter the BUN and creatinine from the blood, leading to acute renal injury. IL-1 and IL-6 will circle through the vascular system and enter the central nervous system (CNS), with the hypothalamus being the destination. The hypothalamus is responsible for maintaining body temperature. The high concentrations of IL-1 and IL-6 within the hypothalamus will cause it to release prostaglandins that will help reset the core body temperature to be higher than normal resulting in fever. The role of IL-1 and IL-6 in increasing the core body temperature is important as the most common initial symptom of COVID-19 is a fever.

Clinical symptoms

The period from onset of COVID-19 symptoms to death ranged from 6 to 41 days with a medium of 14 days; however, this period is dependent on the age of the patient and the status of the patient’s immune system [18]. The incubation period was shorter among patients >70 years of age compared with those under the age of 70 [26]. Furthermore, the risk of infection is notably greater in individuals with pre-existing medical conditions such as asthma, hypertension, diabetes, etc. It has been hypothesized that angiotensin-converting enzyme (ACE) inhibitors or angiotensin receptor blockers (ARBs) may increase the risk of SARS-CoV2 infection and increase the severity of COVID-19 [22]. Contrary to popular belief, ACE inhibitors and ARBS can cause an upregulation of ACE2 receptors, upwards of 3-5-fold. The increased ACE2 receptors allow the viral genome of SARS-CoV2 to be replicated exponentially, causing an increased severity of symptoms. This is one of the primary explanations as to why patients with hypertension are at greater risk of viral infection because the SARS-CoV2 virus will use the ACE2 receptor to enter into the host cell. The respiratory symptoms are more severe in patients with chronic vascular disease (CVD), which might be associated with increased secretion of ACE2 in these patients compared with healthy individuals [36]. The American Heart Association (AHA) released a statement recommending the continuation of these drugs for patients already receiving them for heart failure, hypertension, or ischemic heart disease [22,37]. Because of the increased susceptibility with age and pre-existing conditions, many elderly individuals present with twice the risk of infection as opposed to a healthy 30-year-old individual. The increased presentation of respiratory symptoms within the elderly may also be due to changes in the lung anatomy over time and muscle atrophy leading to changes in the physiological functions of the respiratory system, reduced airway clearance, reduced lung reserve, and reduced defense barrier function [37]. Furthermore, it has been postulated that the number of ACE2 receptors increases over age, thus causing the elderly to be more susceptible than teens even though a number of younger patients may be presenting with similar symptoms. There is also a greater incidence of cardiovascular symptoms within the COVID-19 infected patients, owing to the systemic inflammatory response and the immune system disorders during disease progression [34]. Another proposed mechanism of myocardial injury is that the cytokine storm triggered by an imbalanced response by type 1 and type 2 T-helper cells and respiratory dysfunction and hypoxemia caused by COVID-19, resulting in damage to myocardial cells [38-39]. The common symptoms at the onset of COVID-19 illness are fever, cough, and fatigue, while other symptoms include sputum production, headache, hemoptysis, diarrhea, dyspnea, and lymphopenia [18].

**Figure 5:**
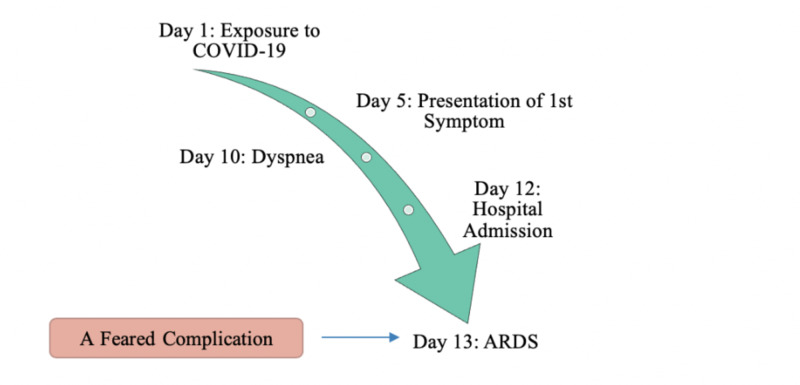
Median observed timeline from presentation of first symptom to ARDS ARDS, acute respiratory distress syndrome

Figure 5 depicts an outline of the median time observed between symptoms [26].

As mentioned previously, the average period between exposure to the presentation of the first symptom is roughly about five days. The initial symptoms of COVID-19 present as being nonspecific symptoms that can be mistaken as a common cold; however, as the replication of the virus increases within the lungs, the severity of respiratory symptoms increases. Respiratory symptoms, most commonly dyspnea, may show up in an infected individual roughly five days after the presentation of the first symptom. Dyspnea, cough, and hemoptysis could be a result of the fibrotic changes that occur within the lungs due to damaged alveolar epithelial cells. In some cases, multiple peripheral ground-glass opacities were observed in subpleural regions of both lungs that likely induced both the systemic and the localized immune response that led to increased inflammation [18-40]. The most common finding upon hospital admission within COVID-19 patients is multiple peripheral ground-glass opacities, indicative of the emergence of respiratory symptoms [40].

The symptoms an infected individual will present with are directly proportional to the strength of the individual’s immune system. In individuals possessing a stronger immune system, symptoms may be milder as the individual’s internal system is able to withhold the cytokine storm and inflammation that occurs. This would be the complete opposite in those with a weaker immune system, such as the elderly. The presence of pre-existing conditions, a high possibility in the elderly population, causes the immune system to weaken, thus leading to the presence of a more severe manifestation of COVID-19. Upon admission to the hospital, which is roughly seven days after the presentation of the first symptom, infected individuals typically present with multiple symptoms ranging from nonspecific symptoms to respiratory symptoms such as hemoptysis, chest pain, dyspnea, etc. [26]. Once admission, health-care professionals will provide symptomatic care and management and, in severe cases, may also put the individual on respirators. Approximately eight days after the presentation of the first symptom, an individual may present with ARDS. Progression to ARDS becomes extremely dangerous for the individual as this is, as portrayed in Figure 5, one of the feared fatal complications of COVID-19. 

Because ACE2 receptors are not only present in the alveolar cells of the lungs, but also enterocytes, patients with COVID-19 may also present with distinct gastrointestinal symptoms. In elderly patients, COVID-19 infects the lower respiratory tract with the potential of leading to fatal pneumonia [41]. Other non-specific symptoms include fever, cough, myalgia, and dyspnea, with or without diarrhea [40-41]. In the second week of infection, it progresses to hypoxemia, difficulty breathing, and ARDS. Patients at this stage may require mechanical ventilation in intensive care units (ICU) in which organisms, such as *Staphylococcus aureus*, *Streptococcus pneumoniae*, *Klebsiella pnuemoniae*, and *Haemophilus influenzae* may cause secondary bacterial pneumonia [41-42].

**Figure 6:**
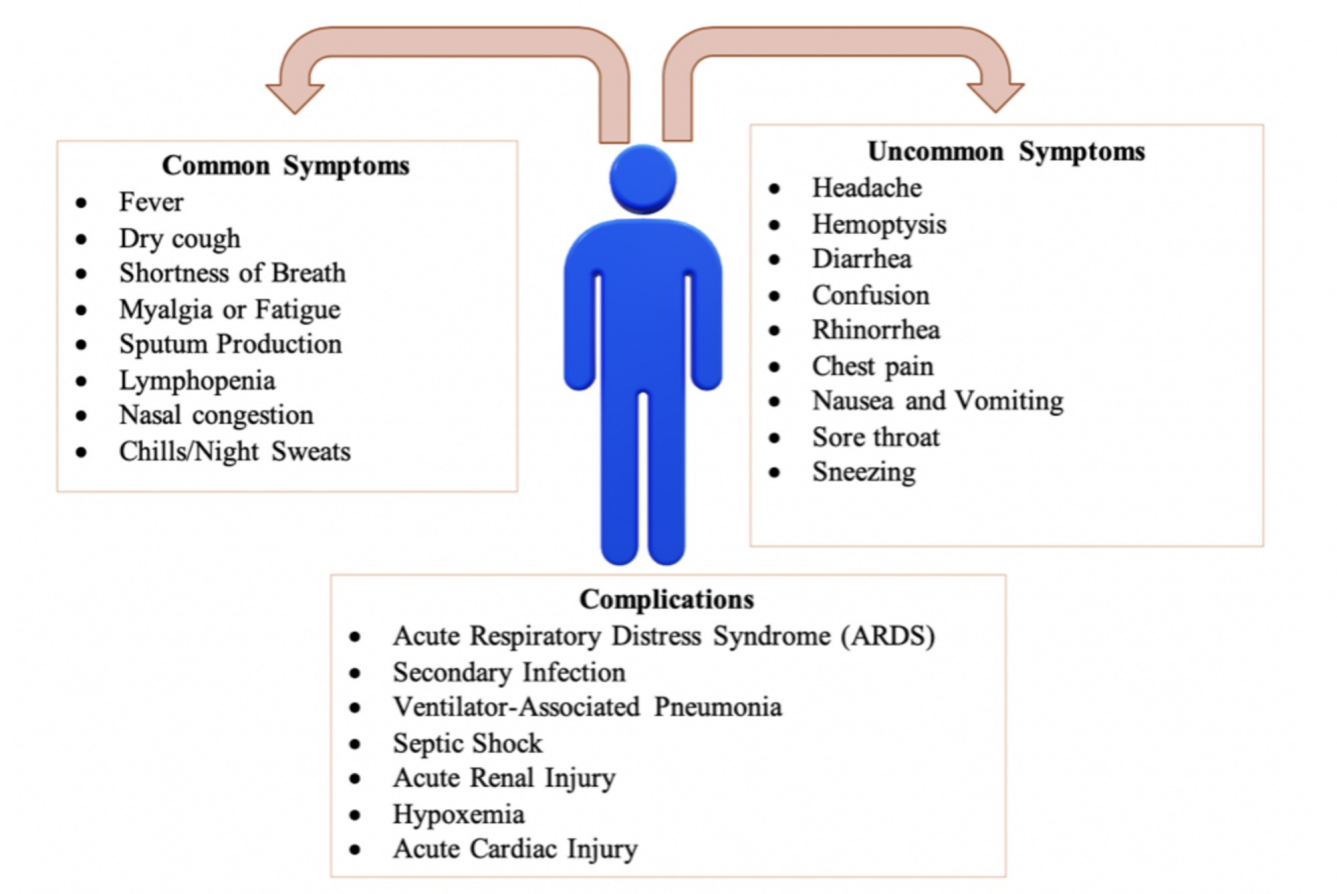
Categorization of the common and uncommon symptoms of COVID-19 patients

Figure 6 is a pictorial representation of a study that was conducted in which researchers reviewed published clinical features, symptoms, and complications amongst the infected patients [43]. For the purpose of categorizing which symptoms are common and uncommon, symptoms that were present in less than 20% of the infected patients were listed as uncommon. The review also lists the most common complications that are seen with infected patients as the severity of COVID-19 shows signs of progression. Complications are more likely to be seen with hospitalized patients, immunocompromised patients, and the elderly population, as mentioned above, and may be fatal. The chances of recovering once any of the listed severe complications have set in become very grim and unlikely.

Clinical management and treatment

As the COVID-19 global pandemic continues to remain severe, there is a greater emphasis on supportive care and symptomatic management while also preventing transmission. Most people that present with mild coronavirus symptoms will recover on their own, and during this period of recovery, they are recommended to isolate themselves for seven days [44]. Social distancing is imperative for individuals to reduce the risk of human-to-human transmission that may occur via asymptomatic carriers or symptomatic individuals. It has been stated that 81% of patients will present with mild to a moderate presentation of COVID-19 with mild symptoms up to mild pneumonia; however, in the remaining 19%, the median time to ARDS ranged from 8 to 12 days and the median time to ICU admission ranged from 10 to 12 days [22,26]. Patients with a mild clinical presentation, such as those with the absence of viral pneumonia and hypoxia, may not initially require hospitalization, and many patients will be able to manage their illness at home [26].

Multiple drugs have been subjected to clinical trials in order to attempt to find a pharmaceutical intervention for COVID-19. Hydroxychloroquine had been considered as a possible therapeutic agent for COVID-19 patients; however, there is limited data on the efficacy and associated adverse events [45]. Hydroxychloroquine is used to treat malaria and rheumatoid conditions such as arthritis, and in various studies, the drugs had demonstrated antiviral activity, an ability to modify the activity of the immune system, leading to the hypothesis that it may have been useful in the treatment of COVID-19 [46]. However, the usage of hydroxychloroquine in COVID-19 has recently been debunked. A study conducted by Rosenberg *et al*. found that among patients hospitalized in metropolitan New York with COVID-19, treatment with hydroxychloroquine was not significantly associated with differences in in-hospital mortality [47]. Furthermore, patients taking hydroxychloroquine were twice as likely to suffer cardiac arrest [46-47]. While the use of hydroxychloroquine has been turned down, several other pharmaceutical interventions are still being investigated such as remdesivir. Remdesivir is a monophosphoramidate prodrug of an adenosine analog that has a broad antiviral spectrum including filoviruses, paramyxoviruses, pneumoviruses, and coronaviruses [48]. Remdesivir is a potent inhibitor of SARS-CoV2 replication in human nasal and bronchial airway epithelial cells [49]. That being said, the clinical and antiviral efficacy of remdesivir in COVID-19 remains to be established [48]. With multiple different pharmaceutical approaches still under research and being pushed through clinical trials, it is imperative to know that no one drug of choice has been narrowed down upon. 

## Conclusions

Originating from a reservoir of bats with pangolins as the presumable intermediate host, SARS-CoV2 binds to ACE2 with high affinity as a virus receptor to infect humans and does so through spike glycoproteins on the surface of its membrane. With the presence of the S glycoproteins on the surface of the virus, coronavirus is able to penetrate the alveolar cells, such as type II pneumocytes, transferring the viral genome for replication within the cell host. Upon the replication of the viral material, it is released from type II pneumocytes and causes a cascade of cytokines to be released. The cytokine storm triggers symptoms such as dyspnea, chest tightness, etc. The initial stages of COVID-19 manifestations present with symptoms that often are confused with that of the common cold such as a fever, myalgias, sneezing, stuffy nose, etc. The appropriate practice of social distancing and isolation when common cold symptoms present is crucial to attempt to reduce the R0. As the infection spirals out of control, those on ventilators are at a greater risk of developing secondary bacterial pneumonia that further complicates the infection and causes conditions such as severe acute respiratory distress syndrome or septic shock, leading to death.
